# Reliability of journal impact factor rankings

**DOI:** 10.1186/1471-2288-7-48

**Published:** 2007-11-15

**Authors:** Darren C Greenwood

**Affiliations:** 1 Biostatistics Unit, Centre for Epidemiology & Biostatistics, University of Leeds, Leeds, UK

## Abstract

**Background:**

Journal impact factors and their ranks are used widely by journals, researchers, and research assessment exercises.

**Methods:**

Based on citations to journals in research and experimental medicine in 2005, Bayesian Markov chain Monte Carlo methods were used to estimate the uncertainty associated with these journal performance indicators.

**Results:**

Intervals representing plausible ranges of values for journal impact factor ranks indicated that most journals cannot be ranked with great precision. Only the top and bottom few journals could place any confidence in their rank position. Intervals were wider and overlapping for most journals.

**Conclusion:**

Decisions placed on journal impact factors are potentially misleading where the uncertainty associated with the measure is ignored. This article proposes that caution should be exercised in the interpretation of journal impact factors and their ranks, and specifically that a measure of uncertainty should be routinely presented alongside the point estimate.

## Background

Journal citation reports are used widely as the basis for assessing research output. They are used by funding bodies to gauge the quality of publications, by researchers to assess which journals they choose to submit manuscripts to, and as a basis for journals to attract new subscriptions and advertising [1]. In addition, current discussion surrounding the future nature of the UK Research Assessment Exercise (RAE) involves greater use of such bibliometric data [2,3]. The journal impact factor is the most widely cited bibliometric tool used to characterise journals. It was originally proposed 50 years ago as a measure of the impact that individual articles have on the research community [4], but it is now more commonly used across all articles published by a journal to provide a measure of a journal's impact on the research community rather than the impact of an individual article [5]. The journal impact factor is thus calculated as the number of citations a journal has received in the last complete year for articles published in the two preceding years, divided by the total number of articles the journal published in the two preceding years. So it gives an average number of citations of published articles, without giving any unfair advantage to the larger or more frequently published journals.

Many journals have taken to quoting their impact factor rank compared with other journals in the same field [1,5]. For example, *Nature Medicine*, with a journal impact factor of 28.9 is currently ranked number one in terms of its impact factor in the field of research and experimental medicine based on the 2005 Journal Citation Reports, whilst *BMC Medical Research Methodology *currently advertises an unofficial journal impact factor of 1.58 based on citations in 2006, which would rank it approximately 45^th ^compared to other journals' citations reported in 2005. There has been considerable discussion regarding the merits and weaknesses of the journal impact factor [1,6-18] but very little comment on the precision with which they are quoted [1,19,20].

The underlying rate at which any particular article is cited is subject to sampling error, as is the underlying rank. These figures are used without regard to the uncertainty attached to their estimation, violating many journals' own guidelines for presentation of point estimates within the research articles that they publish [21]. Previous studies have shown that when confidence intervals are used for performance indicators, especially for ranks, the degree of uncertainty associated with such estimates is such that very little credence can be given to them [22-25]. In this article I have therefore explored the hypothesis that there is similar uncertainty associated with journal rankings based on the journal impact factor. The strength of evidence for the ranking of journals is investigated for the journal citation reports category of "medicine, research and experimental", based on citations in the most recent complete year of Journal Citation Reports, 2005.

## Methods

The number of citations in 2005 to articles published in 2003 and 2004, and the number of articles published in 2003 and 2004 was sourced from the Web of Science [26] for all journals listed under the category of "medicine, research and experimental".

A random effects Poisson model allowing for over-dispersion was fitted within a Bayesian framework using Markov chain Monte Carlo (MCMC) methods in WinBUGS 1.4 [27]. This overcomes problems of small samples by pooling information from each journal and provides more reliable estimates [24,28]. The observed frequency of citations *O*_*i *_for journal *i *in the two years preceding the year the journal impact factor refers to was assumed to follow a Poisson distribution. If *n*_*i *_is the number of articles published by journal *i *during that time period, and *λ*_*i *_is the underlying citation rate per article for journal *i *(the journal impact factor), then we assume the model *O*_*i *_~ Pois(*λ*_*i*_*n*_*i*_), where ln(*λ*_*i*_) = ln(*λ*) + *υ*_*i *_and random effect *υ*_*i *_~ N(0, *σ*^2^) to allow for over-dispersion.

The overall geometric mean citation rate is represented by *λ*. This was given a minimally informative but proper prior ~ N(0.01, 1000). The precision of the random effect (1/*σ*^2^) was given an uninformative gamma prior ~ Γ(0.001, 0.001). In this way the heterogeneity between journals is modelled and the underlying distribution of citation rates can be characterised. Using MCMC it was also possible to rank the parameter realizations from the posterior distribution to give mean or median ranks with associated 95% credible intervals. The credible interval indicates the plausible range of values within which the true journal rank lies and is the Bayesian equivalent of a confidence interval.

## Results

**Figure 1:**
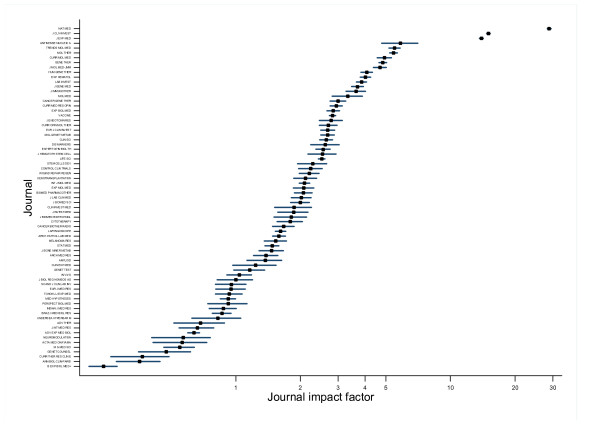
Underlying journal impact factors with 95% credible interval for journals in research and experimental medicine. Source: Web of Science, Thomson Scientific, accessed April 3, 2007.

**Figure 2:**
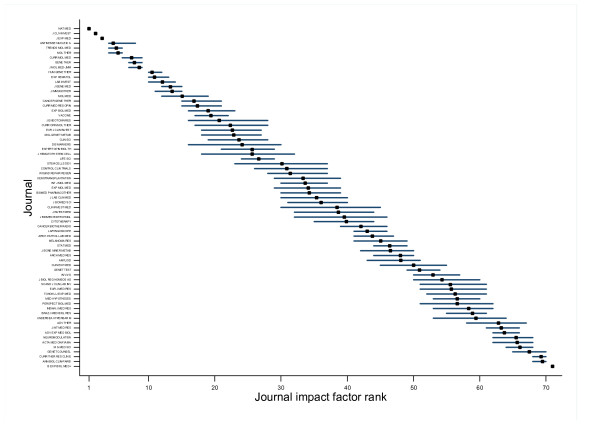
Mean rank of underlying journal impact factor with 95% credible interval for journals in research and experimental medicine. Source: Web of Science, Thomson Scientific, accessed April 3, 2007.

Convergence to a stationary distribution appeared to be achieved after a 25,000 update burn-in. Adequate mixing and convergence was confirmed by assessment of trace plots and Brooks-Gelman-Rubin statistics [29]. This was followed by a further 50,000 updates for each chain to give Monte Carlo error for each parameter of interest less than 5 percent of the sample standard deviation. The underlying citation rate for each journal, *λ*_*i *_(the underlying journal impact factor), taken as the mean over the updates for each parameter, together with the uncertainty associated with its estimation, provided by 95% credible intervals, is shown for research and experimental medicine journals in figure 1. The intervals overlap for a large proportion of the journals. The rates show the usual slight shrinkage whenever a random effects model is used. The mean rank associated with each of the underlying journal impact factors are shown in figure 2. Again, there is considerable overlap of the plausible range of ranks for all journals other than those in the top or bottom few ranks.

Over all journals, the mean width of 95% credible interval for the journal impact factor ranks is 7 places, with the widest plausible range of ranks being 15 places for one journal. The credible intervals for the journals ranked in the top three are narrow and the top journal has a 95% credible interval of (1 to 1). For the middle ranked journals the intervals are somewhat wider, with greater overlap.

## Discussion

The top three journals can be confident that their impact factor rank is fairly precise, with the top journals having no overlap with the lesser journals. For the majority of journals further down the table, the ranking of journals with lower impact factors has less certainty attached to it and their ranking can only be confidently placed within quite a wide range. For example, a journal's impact factor ranking could easily vary by 10 or so positions, without implying any meaningful change in citation performance.

The width of intervals for other research areas will depend in part on the number of citations, reflecting the activity in the research community. For example, for journals in the field of probability and statistics a journal's impact factor ranking could easily vary by 20 or 30 positions, equivalent to ranging over one quarter or one third of the table. Other bibliometric measures, such as the immediacy index, are based on citations in a shorter period of time. These measures will have much wider credible intervals around their estimates, and wide overlapping credible intervals around their ranks.

A total of 19,393 articles were published in the area of research and experimental medicine during the years covered. It was therefore beyond the scope of this project to extract information on each of these individual articles. This additional information would allow variation at the level of the individual article (within journal variability) to be taken into account, thus increasing the width of the credible intervals, and allowing further exploration of the potential problems of taking the journal impact factor to be the mean of a highly skewed distribution [15]. However, by modelling the counts as a Poisson distribution, the confidence intervals are valid for the measure used.

It is now well established that ranking institutional performance such as school examination scores or hospital mortality rates, and ranking individual performance such as a surgeon's success rate, is subject to considerable uncertainty [22-25]. This uncertainty must be taken into account before comparison can be made and inferences drawn [22,23,25]. The Royal Statistical Society has itself published guidelines on the use of such performance indicators, stating that "league tables without demonstration of ranking uncertainty should be avoided and, even with uncertainty incorporated, such tables should be used with considerable caution" [30]. The journal impact factor is analogous to a performance indicator for journals, and as such many of the recommendations for performance indicators could equally be applied to the use of journal impact factors. In particular, a measure of uncertainty should be presented alongside a journal impact factor ranking.

The figures presented appear to show less uncertainty in the ranks, and associated narrower plausible ranges around the ranks, than those investigated to date in the field of institutional performance. This is because of the large number of citations that some journals receive. However, this will depend to a large extent on the research discipline covered by the journal, and some disciplines may demonstrate greater or less uncertainty than the category of research and experimental medicine. For example, in the category of probability and statistics, intervals are much wider, demonstrating greater uncertainty in the estimates.

The implications for the use of bibliometric measures is that decisions based purely on ranks should be avoided. Less emphasis should be placed on small differences in rank order because this may be attributable to random variation. This suggests that arbitrary cut-offs or thresholds should be avoided when making decisions, as there will be very little difference in the underlying citation rate for journals either side of the cut-off. Journals whose impact factors and ranks are broadly similar should be treated similarly, no matter which side of an artificial divide they are placed, because these measures are estimated with considerable uncertainty. Instead, the measure should be kept as a continuous scale so that small differences between journals are associated with similar decisions.

It might be argued that there is no uncertainty associated with the journal impact factor, because it is based on a census of all publications over a given period, and is therefore free from sampling error. However, the main purpose of quoting the journal impact factor is as a performance indicator, drawing inference about the general impact of the journal. This, then, use the journal impact factor as a measure of the underlying rate at which any particular article in the journal is cited. This rate is unknown and must be estimated subject to sampling error. It therefore requires a measure of the uncertainty associated with the estimate.

Many medical journals are signatories to the uniform requirements for manuscripts submitted to biomedical journals [21]. These state that measures of uncertainty, such as confidence intervals, should be presented alongside estimates. This is echoed by most modern textbooks on research methods in medicine. Given that important funding and employment decisions are often based on the estimated impact factors of journals in which researchers publish their work, and on the journal impact factor ranks, an additional useful tool for the researcher would be to know how much uncertainty existed in the bibliometric measure on which they base their decisions. This article therefore proposes that a plausible range, such as a credible interval, be used alongside the estimated journal impact factor and its rank.

This proposal also applies to other journal performance indicators that may be used. For example, the immediacy index is the number of citations received by a journal in the last complete year, divided by the number of articles published by that journal in that year. It therefore gives a measure of how quickly articles from a journal are cited. Like the journal impact factor, it avoids any advantage to larger journals, but it may advantage those that publish more frequently. This measure has more uncertainty associated with it than for the journal impact factor (data not shown) because it is based on a shorter time period. Even if only broad banding of journal immediacy index ranks are used, most journals outside the top ones could not confidently identify whether they were ranked in the top or bottom halves of the table.

## Conclusion

There are strong similarities between the use of journal impact factor ranks and the league tables used to present performance indicators for hospitals and schools [22-25,30]. Both suffer from the potential for comparisons based on ranks to be misleading. The strength of the work presented in this paper is to quantify the lack of precision in journal impact factor ranks and highlight the care that must be taken in their use.

The implication for journals, researchers and advertisers is that only limited confidence should be placed on the ranking of these indicators. Decisions placed on such measures are potentially misleading where the uncertainty associated with the measure is ignored. Journals should follow their own guidelines for presenting data [21] by including a measure of uncertainty when quoting performance indicators such as the journal impact factor or immediacy index.

## Competing interests

The author(s) declare that they have no competing interests.

## Authors' contributions

DCG conceived the study, analysed and interpreted all data. DCG drafted the paper. All authors read and approved the final manuscript.

## Pre-publication history

The pre-publication history for this paper can be accessed here:


